# Fabrication and Characterization of Inhomogeneous Curved Artificial Compound Eye

**DOI:** 10.3390/mi9050238

**Published:** 2018-05-15

**Authors:** Fengli Liu, Xiaolei Diao, Lun Li, Yongping Hao, Zhongyuan Jiao

**Affiliations:** 1 Department of Mechanical Engineering, Shenyang Ligong University, Shenyang 110159, Liaoning, China; xiaoleidiao123@gmail.com; 2 Technology Center of CAD/CAM, Shenyang Ligong University, Shenyang 110159, Liaoning, China; allen.li8910@gmail.com (L.L.); yphsit60@gmail.com (Y.H.); 3 Lovol Heavy Industry Co., Ltd., Weifang 261206, Shandong, China; Zhongyuanjiao110159@gmail.com

**Keywords:** microfabrication, compound eye, polydimethylsiloxane (PDMS), mold

## Abstract

Compared with the conventional compound eye processing method, a new fabrication method—namely, a mold casting method—was presented. This method is simple, low-cost, easy to implement, and can be reused. A bionic compound eye array model with 61 ommatidia arranged inhomogeneously onto a curved surface was fabricated. The curved surface had a radius of 9 mm and a thickness of 0.5 mm. The margin imaging quality was improved significantly by the analysis of light beam focus and the optical imaging properties of the fabricated compound eye. The sub-image of each ommatidium had a high resolution. There was 5% error between the collecting spot brightness and simulation analysis results, which proved that the production method is feasible.

## 1. Introduction

Compound eyes, found in certain arthropods such as insects and crustaceans, are composed of hundreds of “eyelets” within each eye. The image perceived by a compound eye is a combination of numerous sub-images [1]. Inspired by insect compound eyes, research has been conducted to explore bionic compound eyes as a natural solution to the issues of wide-field-of-view [2], high update rate, small volume, and fast motion detection. Bionic compound eyes have recently been used in various applications including inspection information collecting, intelligent control, environmental monitoring, robot navigation, and so on [3].

The current structure of a bionic compound eye consists of a planer and curved structure. While bionic compound eye imaging systems mostly adopt a flat structure design, a number of simple planer compound eyes have been developed [4]. Although the process is simple, the smaller field of view angle limits its application scope and it is difficult to satisfy the requirement of a large field of view. For most fabrication processes, however, the direct generation of optical units onto curved surfaces is a challenge. To enlarge the view angles and decrease the cost of the device, a simple process to form ommatidia on a curved surface is required.

A single curved surface artificial compound eye can solve this problem because its structure is closer to the compound eyes of insects, so its image performance is better and the view angle is larger. However, there are still some problems in its structure design and fabrication. Because the current image detectors are based on a planar structure, it is difficult for margin ommatidia to be focused on such detectors, thus increasing the complexity of ommatidia design and the difficulty of processing this technology. To date, only a few methods had been available for fabricating three-dimensional (3D) structures, for example reconfigurable microtemplating [5], laser lithographic fabrication [6], soft lithography [7,8,9], and the hybrid sol-gel method [10]. One of the existing fabrication methods is transfer printing, in which H.C. Ko provided a planar processing approach to create optoelectronic systems on flat, two-dimensional surfaces in unusual designs that tolerate compression and stretching to large levels of strain to be eometrically transformed to nearly arbitrary curvilinear shapes [11]. Digital apposition cameras were proposed by I. Jung [12]. These devices combine elasto-meric compound optical elements with deformable arrays of thin silicon photodetectors into integrated sheets that can be elastically transformed from the planar geometries in which they are fabricated to hemispherical shapes. Using photodetector arrays on thin elastomeric membranes, they are capable of reversible deformation into hemispherical shapes with radii of curvature that can be adjusted dynamically, via hydraulics [13]. The curving of the ommatidial array along the bendable direction and attachment to a rigid semicylindrical substrate with a radius of curvature of 6.4 mm was performed to build the CurvACE prototype [14]. Soft lithography, which is simple, easy to operate, and can copy the ommatidia structure without deformation compared with the original biological structures, can be used repeatedly. A common method first involves fabricating a planer polydimethylsiloxane (PDMS) concave mold by soft lithography, casting polymethylemthacrylate (PMMA) on the model, and then producing convex compound eye-replicating films by pressing or extruding [15]. This difficulty of this method is in the curing of the PDMS in order to peel off and cut the material to achieve the desired soft mask. Furthermore, in this method it is difficult to control the dipping process and peel off the replicating film. Laser lithographic fabrication was proposed by Pubo Qu. et al. [16], in which a femtosecond laser-enhanced wet etching and casting process is followed by a thermomechanical process to convert the film into a hemispherical surface. He [17] developed a casting and embossing method based on microelectromechanical systems (MEMS) fabrication to make a curved surface artificial compound eye. A planer elastomeric microlens array that can be mechanically stretched to very large extent was presented by Zhengwei et al. [18]. Lei Li and Allen Y. Yi [19], using an ultraprecision diamond machining process, fabricated a microprism array and ommatidia array on curved and flat surfaces, respectively. The electrostatic deformation method is used to fabricate an ommatidia array with different structures by changing the mask shape. However, the process is complicated and the metal wire is likely to experience burnout when the voltage is increased too high. Moreover, the thickness of the PDMS is difficult to control in the spin process and the cost is high. Although biomimetic structures that replicate insect compound eyes can be fabricated by these methods, these techniques require expensive facilities and involve long processing times and complicated fabrication procedures.

The present work focused on improving the image quality for the margin ommatidia and developing a simple fabrication method for a curved surface bionic compound eye. In this compound eye structure, the ommatidia sizes and interommatidia angles were inhomogeneous, and the corresponding parameters were optimized. In this work, the ommatidium on different levels had different focal lengths, which guaranteed that all light spots could be focused on the planar charge coupled device (CCD) and the intensity of the marginal spots were as same as that of the central spots. It is impossible to realize this structure by the existing photoresist melting method. Based on 3D micromachining methods, the mold casting method was proposed to fabricate the compound eye [20,21]. There is only one layer in the compound in this work, which reduced the aligning problem and realized the formation of images on a planar photoreceptor without adding other layers. The advantage of this method is that it is simple, low-cost, and the mold can be used repeatedly. Further, the imaging performance and field of view of a visual system based on this inhomogeneous compound eye were also evaluated, and high resolution for every ommatidium was guaranteed.

## 2. Structure Design for Curved Surface Compound Eyes

**Figure 1:**
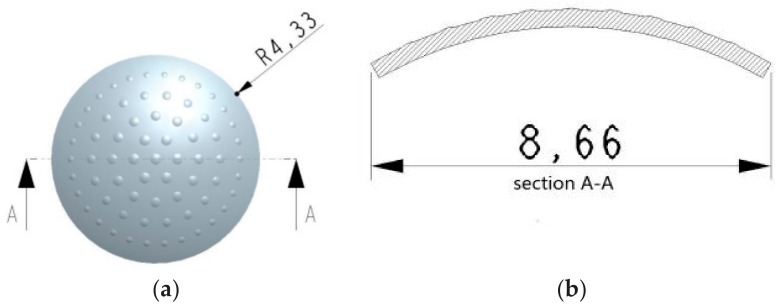
The schematics of a single-layer non-uniform compound eye structure: (**a**) top view; (**b**) section A-A.

The ommatidia on the curved surface which adopted a circular inhomogeneous distribution are shown in Figure 1. The ommatidia located on the same circumference of a circle are assumed to be located on the same level. They have the same focal length and view angle [22]. The imaging performance is improved as the imaging spherical aberration reduced to almost 1% of the initial value. Focal adjustment can be achieved at the same time. So, the problem of margin spherical aberration for each ommatidium is resolved. Furthermore, the design and production are facilitated. The high fill ratio requirement is met.

The distance between adjacent ommatidia is about 1 mm. Taking the cross-section (A-A) through the center of the top ommatidium, as shown in Figure 1, the angles *α_n_* between the ommatidia located on the different levels and the top ommatidium are given in Table 1.

**Figure 2:**
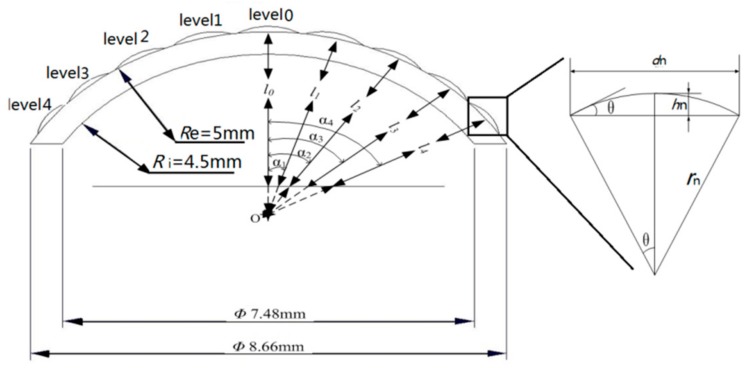
The ommatidia radial arrangement in section A-A.

As the image performances in the same level are same, taking nine ommatidia located on different levels and in the cross-section (A-A) as an example, shown in Figure 2, the corresponding distance *l_n_* between different level ommatidia and the detector is:

(1) $$l_{n}=R_{e}-\frac{R_{e}-l_{0}}{\cos(\alpha_{n})}\;n=1,2,3.......$$

where *R_e_* is the external curved surface radius of the substrate, *l*_0_ is the distance between the top ommatidium and the detector, and $\alpha_{n}$ is the view angle between 0 and *n* level ommatidia. The radii of the internal and external surfaces of the substrate are 4.5 mm and 5 mm, respectively. So, the substrate thickness is 5 mm.

According to the principle of geometry imaging given by Equation (2), the focal length *f_n_* of the ommatidia in *n* level should be equal to *l_n_* and can be calculated by Equation (2):

(2) $$\frac{1}{l_{n}}=\frac{1}{f_{n}}=(n_{0}-1)(\frac{1}{r_{n}}-\frac{1}{R_{e}})\;n=1,2,3.......\;h_{n}=r_{n}\left(1-\cos\theta \right),d_{n}=2r_{n}\sin\theta$$

where *n*_0_ is the refraction index of the ommatidia, *r_n_* is the curvature radius of each ommatidium, *θ* is the tangential angle of each ommatidium, *h_n_* is the height of each ommatidium, and *d_n_* is the width of each ommatidium. The curvature radius *r_n_* for each level ommatidium and corresponding dimension parameters are listed in Table 2.

## 3. Light Tracing

**Figure 3:**
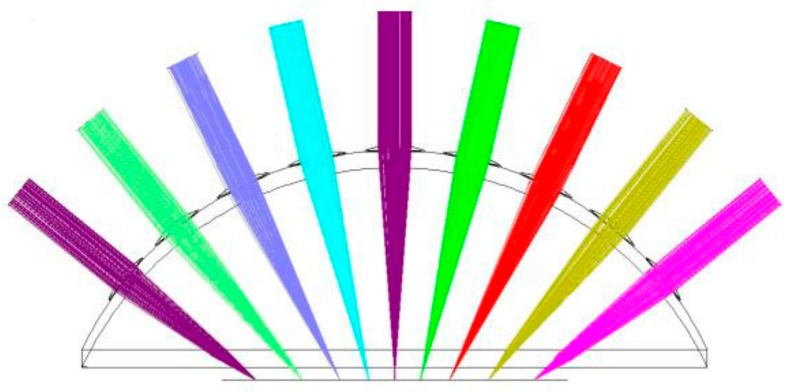
The ray tracing map in section A-A.

**Figure 4:**
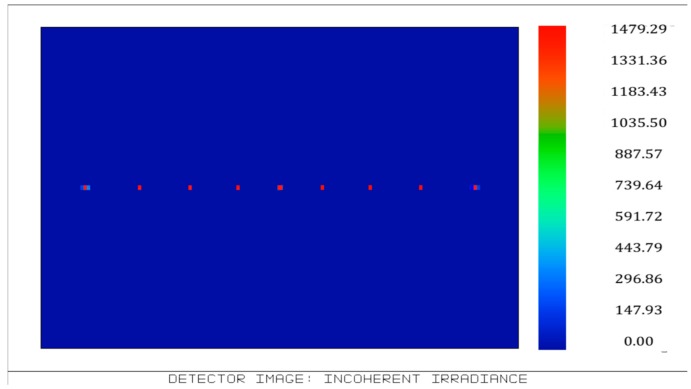
The intensity distribution on the detector in section A-A.

**Figure 5:**
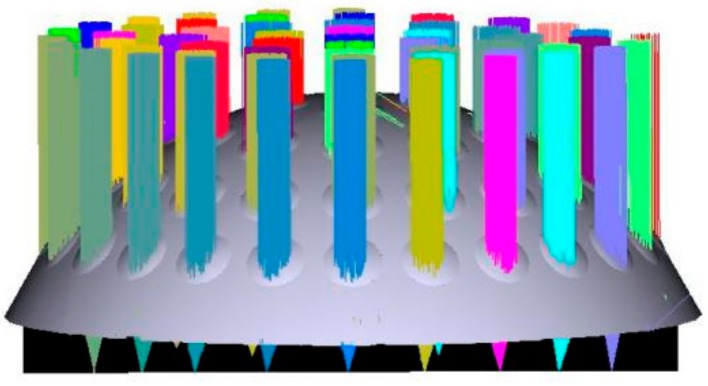
The ray tracing map for all ommatidia.

**Figure 6:**
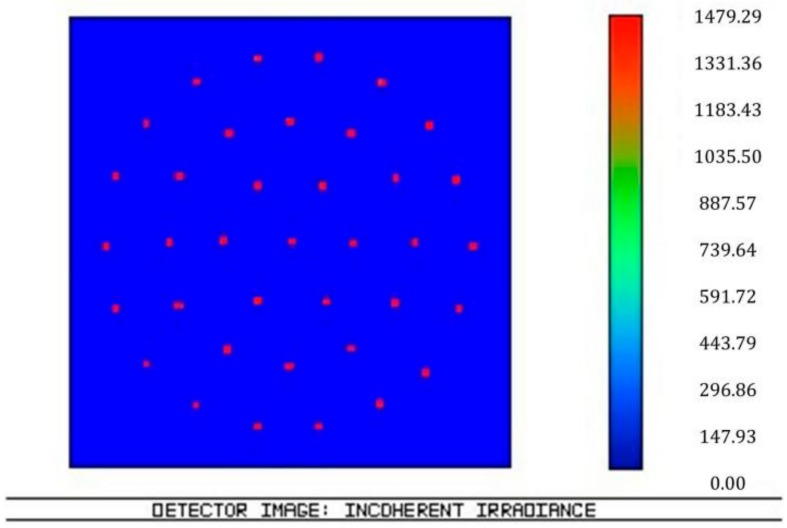
The intensity distribution on the detector for all ommatidia.

**Figure 7:**
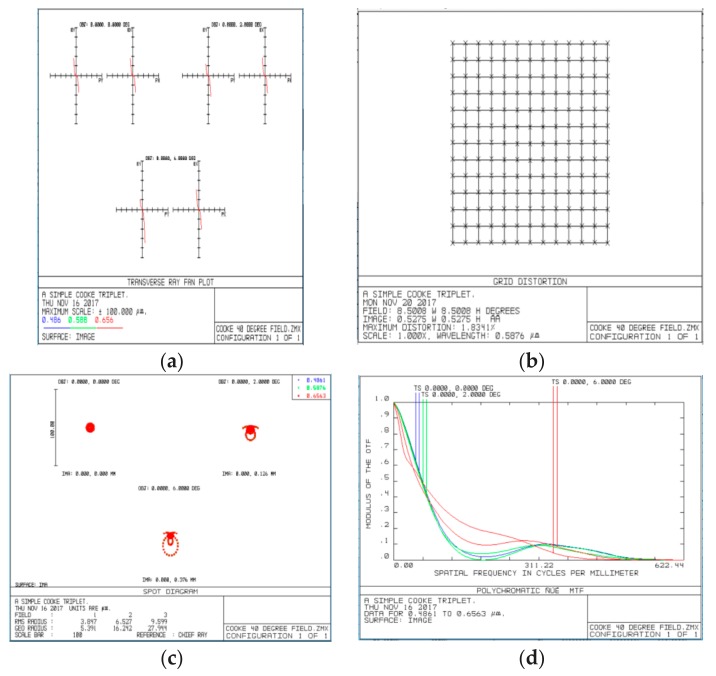
The simulation results: (**a**) ray fan graph; (**b**) grid distortion; (**c**) optical path; (**d**) spot diagram; modulation transfer function.

According to the structure and parameters, light tracing was simulated in Zemax software 2007 (Zemax LLC, Kirkland, WA, USA) and the results are shown in Figure 3, Figure 4, Figure 5 and Figure 6. Figure 3 illustrates the ray tracing map of the nine ommatidia located in different levels in the cross-section (A-A). The ray of light through the nine ommatidia were focused on the plane detector, as shown in Figure 4. The light through these nine ommatidia were completely focused on the detector. The intensity of the marginal spots was as same as that of the central spots, so the marginal image qualities were improved. In order to study whether the whole compound eye focused on the detector, the ray tracing map of all of the ommatidia is shown in Figure 5. The light through all ommatidia focused on the planer detector is shown in Figure 6. Every spot formed on the detector was independent. The sizes of the light spots were uniform and the maximum light intensity reached 1479.29 nt. All of the spots had almost the same intensity, so the images quality from all the ommatidia were uniform. The imaging aberration was small, as shown in Figure 7a. In the grid distortion simulation, shown in Figure 7b, the maximum distortion was 1.8341%, which is less than the human eye’s distortion resolution. The maximum spot radius was 14.442 µm, as shown in Figure 7c. The modulation transfer function proved that the imaging quality was uniform and sharp, as shown in Figure 7d.

## 4. Fabrication of Non-Uniform Artificial Compound Eyes

The curved surface artificial compound eye is close to the compound eye of insects, so its image performance is better while its fabrication is more difficult than the planer artificial compound eye.

**Figure 8:**
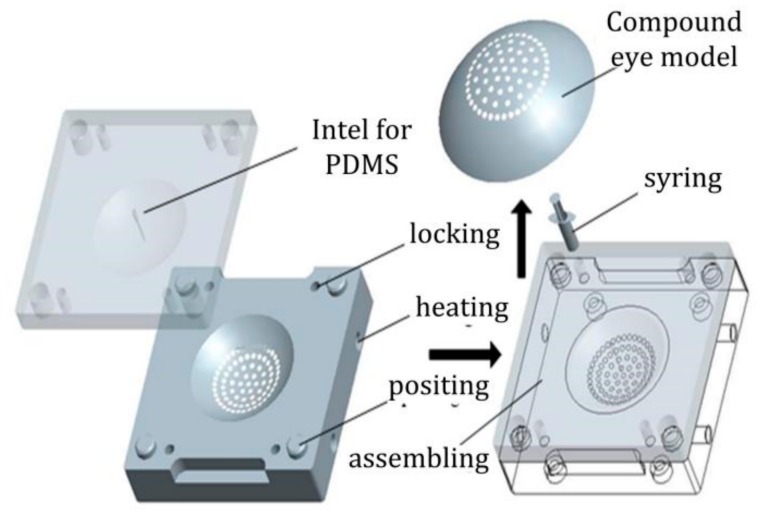
The schematic of curved compound eyes fabrication.

Compared with the existing fabrication methods, a new method for curved compound eyes—mold casting—is put forth in this paper. This method’s advantages include easy operation, low cost, high success rate, and that fact that the module can be used repeatedly. No deformation appears on the finished product. This method includes mask fabrication, copy, and transformation, as shown in Figure 8.

**Figure 9:**
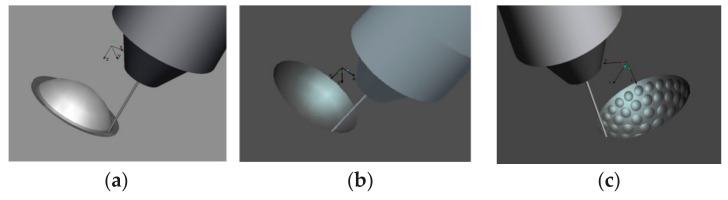
Milling machining process: (**a**) convex mold; (**b**) concave mold substrate; (**c**) compound eye mold.

**Figure 10:**
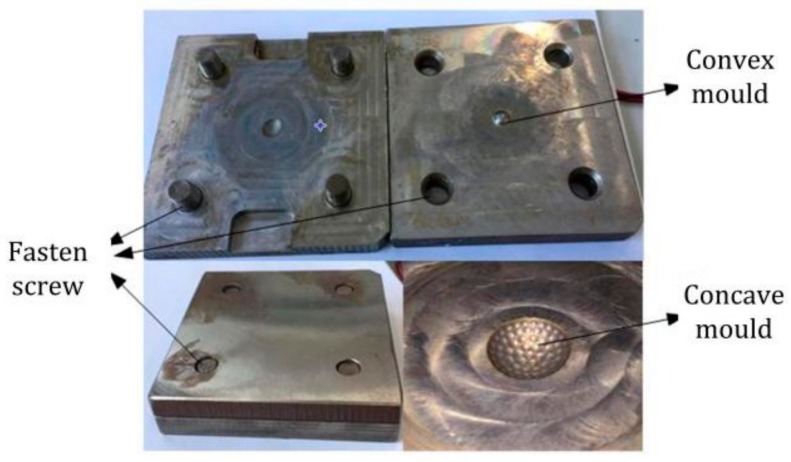
The concave and convex molds.

The first step is the micro-milling of the concave and convex mold on precision five-axis computer numerical control (CNC) machine tools (VMC420, Tengzhou Hoton Machinery Co., Ltd., Tengzhou, Shandong, China). The steps for machining the mold are listed as follows:

(1) The raw material C45 steel was clamped on the precision five-axis CNC machine tools, and the rough machining process was carried out by a vertical spindle milling tool according to the structure and parameters. The convexity in the convex mold and the concavity in the concave mold were obtained respectively by micro-milling, as shown in Figure 9a,b.
(2) The finish machining process was carried by a milling cutter with a smaller diameter ball end. The concave and convex mold for the curved compound eye was finished by the separate feeding of the cutter on every ommatidium’s position in the concavity, as shown in Figure 9c.
(3) Four threaded holes on each corner in the concave and convex molds were tapped to fasten them after pouring the PDMS. The electric resistance wire can be put into the heating hole, which was located in the bottom of the concave mold, if the curing speed needed to be increased. One injecting hole with a 1-mm diameter and an 18.5-mm depth was drilled in the middle of the three ommatidia on the top of the cavity mold. A trace tunnel in the hole was formed after pouring the PDMS. The location of the pouring hole was optimized because it shaded the ommatidia and affected the image performance.
(4) The mold was electrochemically polished, as shown in Figure 10.

The second process involved pouring PDMS (Sylgard 184 silicon elastomer kit, Sigma-Aldrich, Saint Louis, MO, USA) to transfer and copy the graph of the curved compound eyes. PDMS is a kind of macromolecule organic silicon compound with a 1.4 refractive index, 92% light transmittance, low cost, favorable stiction, and chemical inertness. The copy and transferring processes are listed as follows:

(1) Before operation, the module, beaker, and measuring cylinder were cleaned with deionized water by pressure syringe (LSP01-1BH, Longer Precision Pump Co., Ltd., Baoding, Hebei, China) to ensure that no particles remained on them. Then the mold was wrapped in plastic immediately to avoid surface oxidation by contact with the air.
(2) Subsequently, 4 mL PDMS base and 0.4 mL curing agent were mixed in one breaker and a portion of the air bubbles were discharged by standing at least 10 min.
(3) The mixture was injected into the syringe slowly to avoid producing air bubble. The syringe was put into the ultrasonic clearing machine (WN30-BXXW-040AL, Kamson ultrasonic equipment Co., Ltd., Taipei, Taiwan) and vibrated for 40 min to discharge a portion of the air bubbles, then it was left to stand for at least 10 min at room temperature to discharge all of the air bubbles.
(4) The releasing agent (SP-751, Sansaikako, Osaka, Japan) was coated on the mold surface to release the sample from the module easily and thus obtain a smooth sample. Then the mold was fastened by a fastening screw to improve the sealing degree.
(5) A screw syringe needle with a 0.9-mm diameter was put on the syringe and the syringe was inserted into the screw hole in the concave mold. Then the mixture was injected into the module, avoiding the generation of air bubbles.

**Figure 11:**
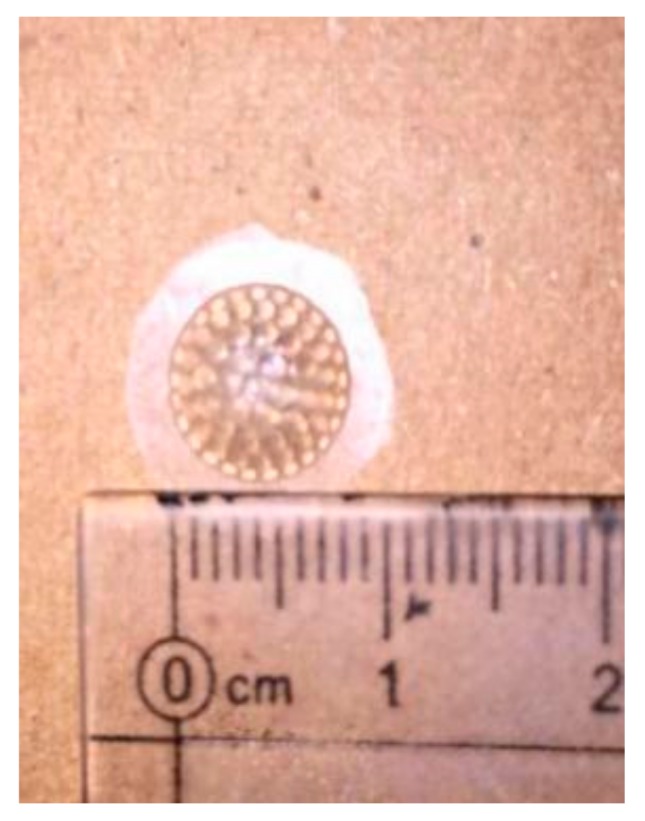
The sample of a bionic compound eye with a radius of 9 mm.

The mixture was then left to stand for at least 30 min to discharge all of the air bubbles. An electric resistance wire was put into the heating hole and the module was heated at least 8 h until the PDMS was cured. A curved compound eye sample was obtained after peeling the sample off of the module, as shown in Figure 11.

## 5. Optical Testing

**Figure 12:**
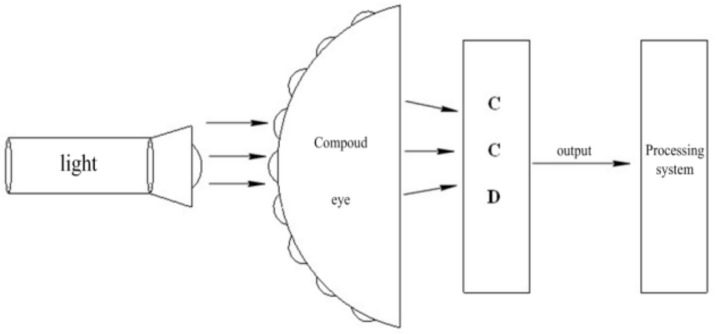
Optical test system bench.

According to the simulation results in Figure 5 and Figure 6, the imaging effect was best when the light was parallel through the central ommatidia and perpendicular to the CCD (M2S130M-H, DO3THINK, Shenzhen, China). Therefore, in the actual experimental testing, we used the parallel light as the incident light. The finished compound eyes were subjected to spotlight effect testing in order to test their optical characteristics. The test platform consisted of a light source, compound eyes, CCD, and computer monitor, as shown in Figure 12.

**Figure 13:**
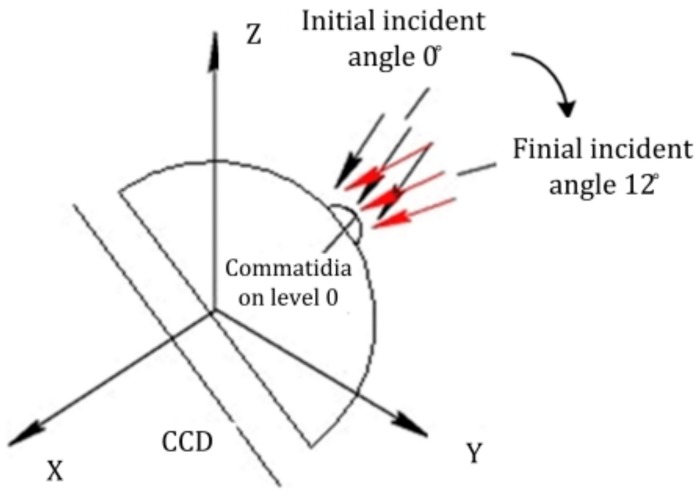
Adjustment of the light incident angle from 0° to 12°.

**Figure 14:**
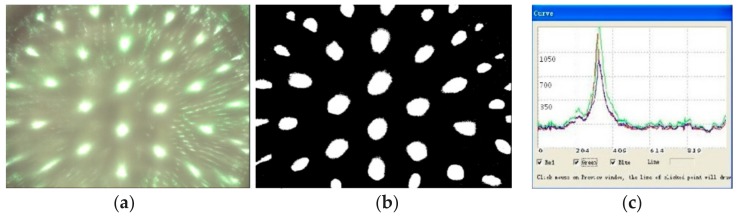
The spot image of a compound eye: (**a**) spot picture; (**b**) binarized diagram; (**c**) spot graph.

The curvature error among these fabrication errors was the worst impact factor for the focusing of the CCD. The simulation results were good when the light was cast vertically in relation to the CCD (when the incident angle was 0°), as shown in Figure 5 and Figure 6. In the testing, the imaging quality was not as clear as the simulation results in Figure 5 under the same situation due to the impact of the existing fabrication curvature error. In order to achieve a comparatively sharp image, we adjusted the incident angle of the parallel light from 0° to 12° to obtain the maximum light intensity on the CCD, as shown in Figure 13. Every spot of each ommatidium was cast on the photosensitive area on the CCD [23,24,25,26]. The light spots image was recorded, as shown in Figure 14. 

Compared with Figure 6, the actual image in Figure 14a is not compatible with the simulation results. the fabrication errors were inevitable because the compound eye mold was fabricated by the five-axis CNC machine tools, so the errors were transmitted to the sample. The image of facula in Figure 14a was not clear, so the image was subjected to binarization processing to obtain a sharper facular image, as shown in Figure 14b. The maximum facula brightness of the top ommatidium was 1400 nt, achieved by analyzing the red, green, and blue light as shown in Figure 14c. There was 5% relative error compared with the formula result of 1479.29 nt, as shown in Figure 6.

**Figure 15:**
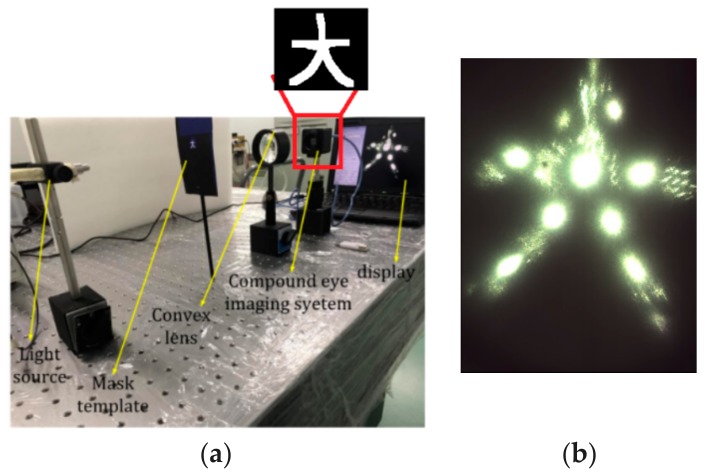
Schematic illusion of optical characteristics of a compound eye: (**a**) experimental setup; (**b**) image on planar screen.

In order to further study the imaging results, we adopted another object to conduct imaging experiments, as shown in Figure 15a. Figure 15b shows an image projected on a planar screen and obtained at three distances (9 cm between light source and object, 3.5 cm between object and convex lens, 1.8 cm between convex and compound eyes) along the optical axis. The images obtained from the margin ommatidium on level 4 were as sharp as those from ommatidia on other levels. The facula brightness of each ommatidium was uniform, as shown in Figure 14. The object imaging in Figure 15 had higher integrity and the image quality was better. Although the image had not undergone binarization processing, the light showed a more uniform focus from the center to the edge, as well as a more homogeneous intensity throughout the image and reduced geometric distortions.

## 6. Conclusions

In this study, we fabricated biologically inspired curved compound eyes, on which 61 ommatidia were arranged inhomogeneously. The structure was designed and the parameters of the ommatidia on different levels were optimized. In the light tracing simulation, the light through all ommatidia focused on the planer detector, and every spot formed on the detector was independent. The high resolution for each margin ommatidium was guaranteed. A new method for curved compound eyes, namely, mold casting, was demonstrated in this paper. This method’s advantages include easy operation, low cost, high success rate, and the fact that the module can be used repeatedly. The finished product was obtained without deformation. The finished compound eyes underwent spotlight effect testing, and the relative error was 5% compared with the formula result. The image quality from all ommatidia were uniform. The images obtained from margin ommatidium on level 4 were as sharp as those from ommatidia on other levels.

## Figures and Tables

**Table 1:** The angle *α_n_* between ommatidia located on the different levels and the top ommatidium.

Level	0	1	2	3	4
$\alpha_{n}$ (°)	0.0	14.0	28.0	41.0	52.0

**Table 2:** Basic dimensions of all levels ommatidia (Unit: mm).

Parameter	Lever 0	Lever 1	Lever 2	Lever 3	Lever 4
Radius (*r_n_*)	1.2	1.19	1.16	1.11	1.03
height of the ommatidia (*h_n_*)	0.24	0.23	0.19	0.15	0.13
focal length	3.44	3.39	3.23	2.93	2.47
